# Moderating Effect of Community and Individual Resilience on Structural Stigma and Suicidal Ideation among Sexual and Gender Minority Adults in the United States

**DOI:** 10.3390/ijerph192114526

**Published:** 2022-11-05

**Authors:** Jennifer R. Pharr, Lung-Chang Chien, Maxim Gakh, Jason D. Flatt, Krystal Kittle, Emylia Terry

**Affiliations:** 1 Department of Environmental and Occupational Health, School of Public Health, University of Nevada, Las Vegas, NV 89119, USA; 2 Department of Epidemiology and Biostatistics, School of Public Health, University of Nevada, Las Vegas, NV 89119, USA; 3 Department of Social and Behavioral Health, School of Public Health, University of Nevada, Las Vegas, NV 89119, USA

**Keywords:** structural stigma, sexual and gender minority adults, suicidality, community resilience, individual resilience: laws and policies, transgender sports bans

## Abstract

Background: Structural stigma in the form of discriminatory laws and policies impacts the mental health of sexual and gender minorities, especially with regard to suicidality. However, this relationship could be moderated by resilience. The past two years has brought anti-SGM legislation, particularly transgender sports bans, at the state level in the United States into focus. This study aims to understand if the relationship between familiarity with transgender sports bans (proposed or enacted) and suicidality was moderated by individual or community resilience. Methods: This was a cross-sectional study of survey data collected from a national sample of 1033 SGM adults in the United States between 28 January and 7 February 2022. Univariate and multivariate moderation analyses were used. Results: In the univariate analyses and the final model, community resilience moderated the relationship between structural stigma and suicidality (*p* = 0.0002); however, individual resilience did not (*p* = 0.0664). Conclusion: Interventions to bolster community resilience may attenuate the negative mental health impacts of structural stigma and are warranted, along with concerted efforts to minimize structural stigma in the form of discriminatory laws and policies targeting people who are SGM.

## 1. Introduction

Sexual and gender minority (SGM), or people who have lesbian, gay, bisexual, transgender, queer, intersex, and other non-cisgender and non-heterosexual identities (LGBTQIA+), both in the United States (US) and internationally, experience mental health disparities compared with their cisgender (not transgender)/heterosexual peers, especially related to suicidality [1,2,3]. A recent study examining suicidality among sexual minorities in the US found that 4% of heterosexual men and women had suicidal thoughts during the past 12 months compared with 11.6% of gay men, 11.0% of lesbians, 17.4% of bisexual men, and 19.9% of bisexual women [2]. Suicidal ideation tends to be higher among younger SGM people [4]; 45% of SGM youth consider suicide each year [5]. A meta-analysis of studies from 10 countries found that SGM youth and young adults were 3.7 to 5.9 times more likely to attempt suicide than their non-SGM peers [6]. Suicide among SGM people is an international concern.

Stigma associated with having a sexual orientation or gender identity that falls outside the cisgender/heterosexual normative culture contributes to increased suicidality in this population [7]. One form of this stigma, structural stigma, can take the form of discriminatory laws and policies and is detrimental to the health and well-being of SGM people globally [8,9,10,11]. Places with higher measures of structural stigma see increased mental distress, suicidality, mortality, social isolation, and a lower quality of life for people who are SGM [12,13,14,15,16,17,18]. Conversely, moving from a place of higher structural stigma to a place of lower structural stigma is associated with decreased depression and suicidality in this population [15]. Specifically, lower structural stigma is associated with a low risk of suicidality among sexual minority men, sexual minority adolescents, and transgender adults [13].

Some research has shown that familiarity with and anticipation of structural stigma also impacts mental health. Horne and colleagues found that familiarity with a referendum proposing to remove legal gender-based protections was associated with increased referendum-associated anxiety, which in turn, was associated with increased levels of depression pre-election [16]. After the referendum failed, retaining gender-based protections, depression, anxiety, and referendum-related anxiety were all significantly lower than before the election. The anticipated structural stigma from losing gender-based legal protections reveals the potential mental health impacts in the SGM community of removing legal protections [19]. Relatedly, Williams and colleagues found that anticipated discrimination was indirectly associated with worse psychological distress through reduced self-compassion and self-esteem as well as indirectly associated with worse self-reported health through reduced social support [20]. To further understand the anticipated structural stigma among SGM adults after the 2016 election in the US, Fredrick and colleagues conducted a qualitative study of SGM adults [19]. Four major themes emerged: “(1) anticipated negative consequences of specific anti-SGM political figures, (2) concerns about the loss of existing SGM rights, (3) fear of new anti-SGM policies, and (4) fears of vulnerability related to limited existing protections.” [19] (p. 348).

Fear and the anticipation of stigma have been realized by many SGM adults in the US as a result of state-level legislative efforts. As of April 2022, over 300 state-level anti-SGM bills had been introduced across 36 US states [21]. While many of these bills focus on the broader SGM community, an important subset focuses on limiting the rights of transgender persons. One category of anti-transgender bills—transgender sports bans—has received a great deal of media attention [22]. State-level transgender sports bans in the US (adopted or proposed) attempt to limit access to sports participation for transgender athletes at the youth, high school, and collegiate levels. Transgender sports bans generally try to require athletes to participate in gender-based sports consistent with the gender assigned to them on their birth certificates; many do not allow athletes to participate based on their gender identities. Between 2020 and June 2022, 18 US states enacted transgender sports bans.

Transgender sports bans are not just prevalent in the US; they are an international phenomenon. Transgender sports bans outside the US focus more specifically on elite athletes and a global scale. For example, in June 2022, FINA, the international swimming federation, banned transgender women from competitions if they had not started medical treatments to suppress the production of testosterone before experiencing any part of male puberty beyond stage 2 on the puberty Tanner Staging or by age 12, whichever came later, and unless they continuously maintained testosterone levels below 2.5 nmol/L [23]. The Human Rights Campaign has argued that meeting this requirement is unrealistic and effectively impossible for many transgender female athletes who live in US states or countries that restrict gender-affirming care [24,25,26,27]. In addition, International Rugby banned transgender women from the competition, and the International Cycling Union imposed the testosterone restriction of 2.5 nmol/L for transgender women cyclists in June 2022 [28].

National and international transgender sports ban debates and targeting a subgroup of SGM people through transgender sports bans may open the door for additional anti-SGM legislation, restrict access or deny services to SGM people, and affect mental health [29,30,31]. Transgender sports bans, if adopted, confirm SGM adults’ concerns by creating new anti-SGM policies and depriving the rights of a subgroup of the SGM community. Our previous research revealed that SGM adults familiar with transgender sports ban legislative efforts reported higher levels of suicidality than those who were not familiar [11]. This finding may result from further anticipated structural stigma, concerns about the loss of rights, or additional anti-SGM laws and policies in the US.

Although structural stigma in the form of anti-SGM laws and policies adversely impacts mental health, resilience can mitigate the adverse mental health outcomes associated with stigma, especially among SGM people in the US and internationally [32,33,34,35]. Resilience is described as the ability to survive, even thrive, in the face of stigmatizing events, as it buffers or attenuates the strength of association between risk (e.g., structural stigma) and outcome (e.g., suicidality) [36,37]. A scoping review examining the use of resilience to understand health outcomes among SGM people identified that there is no universally agreed upon definition of resilience and no specific tool to measure it for the SGM community [38]. The authors’ advocate for a broader definition of resilience that accounts for both individual level factors (e.g., a set of inherent intrapersonal traits like self-efficacy, self-esteem) and broader interpersonal, community, and environmental factors (e.g., perceived social support, social connectedness) [38].

Individual resilience is characterized by individual qualities or dispositional attributes that may help or hinder how a person responds to stigma. These qualities and attributes include self-esteem, self-efficacy, self-liking, social adeptness, cheerful mood, good communication skills, flexibility in social matters, and the ability to uphold daily routines and plans [39]. Individual resilience is not only associated with the mental health of SGM adults, but it also moderates the relationship between adverse childhood experiences and mental health outcomes [40]. Most research on resilience in the SGM community focuses on individual resilience, and there are calls for integrating individual and community resilience concepts in future research [41,42].

Community resilience is the perception that a person has of their access to external support from family and friends. It provides a person the sense that they can overcome challenges and obstacles because of their social networks [39,43]. Research has found that community resilience in the form of connection to the LGBT community is negatively associated with stress among SGM adults [44]. Additionally, research demonstrates that resilience in the form of peer support has a moderating role in the relationship between stigma and psychological distress among transgender persons and between enacted stigma and depression among young men who have sex with men [45,46]. Qualitative research has revealed that social support in the form of formal support or support from meaningful others (e.g., partner, family, friends) is a suicide protective factor [47]. In this study, we defined community resilience based on social resources, focusing on this broader factor that is external to the person rather than an inherent, intrapersonal trait.

Our previous research found an association between familiarity with adopted or proposed transgender sports bans and suicidality among SGM adults even after interpersonal and individual stigma mediated this relationship [11]. Other research shows that individual and community resilience can attenuate the association between stigma and mental health issues among SGM people.

The purpose of this study was to better understand the relationship between familiarity with structural stigma and suicidal ideation by examining the influence of resilience. Based on the literature, discussed above, we hypothesized that individual resilience would attenuate the association between familiarity with structural stigma and suicidal ideation. Additionally, we hypothesized that community resilience in the form of social support would attenuate the relationship between familiarity with structural stigma and suicidal ideation. This study will build knowledge of resilience by utilizing the broader definition of resilience, advocated for by Colpitts and Gahagan, in the analysis to include both individual and community resilience in a multiple moderation model [38].

## 2. Materials and Methods

### 2.1. Study Design and Data Collection

This cross-sectional survey was conducted between 28 January and 7 February 2022, with 1033 adults who identified as SGM from across the US, including Washington, DC. Qualtrics Research Marketing Team participated in recruitment and data collection through an online survey. Participants were recruited through multiple avenues, including apps, games, social media platforms, and Qualtrics’ dashboard-type system. Details about Qualtrics’ project stages are available at https://www.qualtrics.com/panels-project/ [48]. Individuals had to identify as SGM and be at least 18 years old to participate. Eligible participants were given incentives per terms and conditions set forth by Qualtrics and its data collection partners [48]. Participants were provided an electronic informed consent before beginning the survey. If participants agreed to participate, they could start the survey. If they declined, the survey was programmed to terminate automatically. This study was deemed exempt by the University of Nevada, Las Vegas Institutional Review Board because no identifiable information was collected.

### 2.2. Measures

#### 2.2.1. Independent Variable—Structural Stigma

Participants were asked about their familiarity with state-level transgender sports bans by using the following question: “How familiar are you with state-level transgender sports bans being proposed or passed in several states across the US?” [11]. Available responses were not at all familiar, somewhat familiar, familiar, and very familiar. This variable was dichotomized with “not at all familiar” and “somewhat familiar” grouped as “not familiar”; and “familiar” and “very familiar” grouped as “familiar”.

#### 2.2.2. Dependent Variables

The Suicidal Ideation Scale (SIS) was developed by Rudd in 1989 and is a 10-item questionnaire that assesses the presence or absence of suicidal thinking as well as the intensity of those thoughts [49]. Participants are asked to respond to a series of questions using a 5-point Likert scale (1 = never, 2 = infrequently, 3 = sometimes, 4 = frequently, and 5 = always). The scores for the ten questions are summed to compute the SIS score, which ranges from 10 to 50, with a higher SIS score representing a greater suicidal ideation [49]. The SIS has demonstrated high internal consistency (Cronbach’s alpha = 0.91), construct validity for self-harm (r = 0.83, *p* < 0.001), and item-total correlations (rs = 0.45–0.74) [49].

#### 2.2.3. Moderators

The Resilience Scale for Adults was originally developed by Hjemdal and colleagues in 2001 and has been modified into a 33-item instrument to measure resilience across six dimensions: Perception of the Self, Planned Future, Social Competence, Family Cohesion, Social Resources, and Structured Style [50]. Cronbach’s α for subsections ranges from 0.67 to 0.81 and 0.88 for the total score. Test–retest Pearson correlation ranges from 0.73 to 0.80 for subsection and 0.84 for the total score [51]. We selected the Resilience Scale for Adults over other scales measuring resilience because it includes both individual factors (e.g., self-efficacy, self-esteem) and broader interpersonal and community level factors of social resources; measuring both individual and broader factors is advocated by Colpitts and Gahagan [38]. Item responses range from 1 to 7, and mean scores within each subscale are calculated. Higher scores indicate higher levels of resilience. For this study, we summed the following subscales and categorized the summation as individual resilience because they measure personal dispositional attributes: perception of self (e.g., confidence in one’s own ability, self-efficacy, self-esteem, self-liking), structural style (e.g., follows routine, organized, having clear goals and plans), and social competence (e.g., self-perception of flexibility in social interactions, feeling at ease in social settings, presences or absences of a prosocial interactional style, ability to establish friendships) [39,52]. We also used the social resources subscale, which was categorized as community resilience because it measures self-perception of external support (e.g., social support, someone to turn to for help) [39,52].

#### 2.2.4. Confounders

We included the following confounders and provided descriptive statistics about them for our sample: sexual orientation, gender identity, age, education, employment, income, marital status, race, and ethnicity. The question used to gather sexual orientation data was: “What is your current sexual orientation?” (Check all that apply). Answer options were lesbian, gay, bisexual, queer, questioning, asexual, and straight/heterosexual. Participants that selected more than one sexual orientation were recoded as “multiple sexual orientations.” Because having all categories of sexual orientation caused the singularity problem, leading to non-unique solutions in each model, categories other than lesbian, gay, or bisexual were grouped as ‘other.’ The question used to gather gender identity data was: “What is your current gender identity?” (Check all that apply). Answer options were female; male; trans man, trans male; trans women, trans female; genderqueer; gender non-conforming, gender non-binary. Participants who selected two or more gender identities were recoded as “multiple gender identities.” Because having all categories of gender identity caused the singularity problem as well, leading to non-unique solutions in each model, categories other than females and males were grouped as ‘other’.

### 2.3. Statistical Analysis

The moderation analysis was performed to examine whether individual and community resilience were significant moderators in the relationship between structural stigma and suicidal ideation. The two resilience measures were first analyzed separately in the univariate moderation model, expressed as a linear regression of suicidal ideation predicted by structural stigma, individual or community resilience, and the interaction between structural stigma and moderators. Then, we analyzed both resilience measures simultaneously in the multivariate moderation model, expressed as a linear regression of suicidal ideation predicted by structural stigma, individual resilience, community resilience, and two interactions between structural stigma and each moderator. Besides identifying whether individual or community resilience significantly moderated the effect of structural stigma on suicidal ideation, we further examined a follow-up test to determine whether the distribution of individual or community resilience affected suicidal ideation by probing the interactions with the spotlight analysis [53]. The values of each moderator used for probing the interaction were a standard deviation below the mean, the mean, and a standard deviation above the mean, representing the low, medium, and high levels of a moderator.

Both univariate and multivariate moderation models were estimated through the inference of linear regression, including the 95% confidence interval (CI) and *p*-value in each estimated coefficient. Data were cleaned, managed, and analyzed by SAS v9.4 (SAS Institute Inc., Cary, NC, USA). The type I error was set to 5%.

## 3. Results

### 3.1. Participants’ Characteristics

Demographic characteristics in Table 1 show that most reported not being familiar with structural stigma (67.37%); bisexual orientations (46.97%); being female (55.46%), White (74.00%), and Non-Hispanic (85.39%); having some college, no degree, or associate degree (35.54%); being single (46.05%) and employed (48.45%); and having an annual income less than $20,000 (34.03%). The average was 18.36 (standard deviation [SD] = 10.57) in suicidal ideation, 85.48 (SD = 22.21) in individual resilience, 33.74 (SD = 9.37) in community resilience, and 38.56 (SD = 15.72) in age. In particular, the means and SDs of individual resilience and community resilience further determined the moderator values of low (63.51 & 24.45), medium (85.70 & 33.82), and high (107.89 & 43.19) levels for probing the interactions.

### 3.2. Univariate Moderation Analysis

Both univariate moderation models were statistically significant (*p*-values < 0.0001), with an R^2^ of 28.69% and 29.44%, respectively. Table 2 shows that individual resilience was negatively associated with the suicidal ideation score, where a one-point increment of individual resilience significantly reduced the suicidal ideation score by −0.20 points (95% CI = −0.23, −0.26; *p*-value < 0.0001). However, individual resilience was not a significant moderator because its interaction with familiarity with structural stigma was not statistically significant, with a *p*-value of 0.9550. When community resilience was tested as a moderator of familiarity with structural stigma, it was significantly and negatively associated with the suicidal ideation score (estimate = −0.37; 95% CI = −0.45, −0.30; *p*-value < 0.0001). Moreover, community resilience was considered a significant moderator because it significantly interacted with familiarity of structural stigma with a *p*-value of 0.0018.

### 3.3. Multivariate Moderation Analysis

In the multivariate moderation model with individual resilience and community resilience, the R^2^ increased to 33.77%, slightly higher than the R^2^ of the two univariate moderation models. Both resilience measures were significantly and negatively associated with the suicidal ideation score with *p*-values < 0.0001 (Table 2). Structural stigma in the form of familiarity with proposed or adopted state-level transgender sports bans was also significantly associated with suicidal ideation. Participants familiar with this form of structural stigma had a significantly higher suicidal ideation score than those not familiar with it by 9.96 points (95% CI = 4.53, 15.40; *p*-value = 0.0003). Community resilience still significantly moderated the association between familiarity with structural stigma and suicidal ideation score with a *p*-value of 0.0002. Individual resilience neared significance, although it did not reach significance as a moderator in the final model with a *p*-value of 0.0664.

By probing the two interactions in the multivariate moderation model, we found that participants familiar with this form of structural stigma had significantly higher suicidal ideation than those not familiar with it in all combinations (low, medium, and high levels) of the two moderators, except for low individual resilience with high community resilience (Table 3). When individual resilience was low, the difference in suicidal ideation decreased from 6.46 points (95% CI = 4.53, 8.40) to 0.73 points (95% CI = −2.16, 3.61) as community resilience increased from low to high. This decreasing trend was also found for the medium and high levels of individual resilience. Meanwhile, regardless of the level of individual resilience, the suicidal ideation score difference was smaller among participants with and without familiarity of this form of structural stigma when the level of community resilience increased. In contrast, when community resilience was low, the difference in suicidal ideation score increased to 9.24 points (95% CI = 6.30, 12.19) when individual resilience was high. The increasing trend also appeared in medium and high levels of community resilience, indicating that participants with familiarity with this form of structural stigma had higher suicidal ideation than those without familiarity with structural stigma when individual resilience increased.

## 4. Discussion

Our findings suggest that participants who were familiar with the transgender sports ban legislation, representing a form of structural stigma, had higher suicidal ideation scores than those who were not familiar with such legislation This is important because SGM people are already at an increased risk for suicide and suicide ideation compared to non-SGM people. The increased risk for suicide and suicidal ideation is likely due in part to structural stigma in the form of anti-SGM laws and policies, which in this study took the form of adopted or proposed legislation attempting to codify transgender sports bans.

Our finding is consistent with other research linking structural stigma to suicidal ideation among SGM people in the US and internationally [13,15,54]. For instance, a qualitative study of SGM people who had attempted suicide found that recurrent exposure to anti-SGM structural stigma was a source of emotional pain that lowered thresholds for suicidal action [7]. SGM participants who lived in communities with higher anti-SGM structural stigma experienced loneliness and fear of rejection. This pain contributed to suicide attempts as a way to escape the pervasive anti-SGM social norms they experienced in their communities [7].

Other studies also suggest that structural stigma formalized through legislative and policy proposals is linked to suicidal ideation among people who are SGM. A recent study of suicidal ideation among SGM people in Taiwan before and after two referendums to restrict marriage to a union between one man and one woman found higher rates of suicidal ideation after the referendums than before [55]. The anti-same-sex referendums were one of the first times that people in Taiwan expressed their attitudes toward homosexuality and same-sex marriage through a formal policy mechanism like a referendum. After the referendums, SGM people perceived higher rates of unfavorable attitudes toward homosexuals and same-sex marriage within Taiwanese society and among their heterosexual friends [55]. These unfavorable attitudes were positively associated with suicidal ideation among SGM people.

Our findings, coupled with those of other studies, demonstrate the influence of law and policy, even when just proposed, on shaping perceptions of structural stigma and mental health outcomes, such as suicide attempts and suicidal ideation, among SGM people. Law and policy can serve as vehicles for structural stigma. However, protective laws and policies can also buffer structural stigma. Studies have found that the passage of SGM-inclusive laws is associated with reduced suicide attempts among SGM people [54]. This finding suggests a need to reduce structural stigma through SGM inclusive laws and policies as a pathway to target mental health outcomes among SGM people.

This study also examined the moderation effect of resilience on the association between familiarity with structural stigma and suicidal ideation. In our final model, community resilience moderated the association between structural stigma and suicidal ideation, while individual resilience did not appear to have this effect. Our finding that individual resilience did not moderate the association between structural stigma and suicidal ideation adds to a complicated literature about the role of individual resilience as a moderator. Other studies have demonstrated a moderating effect of individual resilience on suicidal ideation and health within certain subgroups of the SGM community but not others. For instance, Miceli and colleagues found that individual resilience moderated the association between minority stress and suicidal ideation for bisexual people with a mental health diagnosis but not for bisexual people without a mental health diagnosis [56]. Additionally, Woodford and colleagues found a significant interaction between resilience and interpersonal microaggressions on suicidal ideation among sexual minority students but not among gender minority students [57]. Baiocco and colleagues found that individual resilience moderated the association between discrimination and health among Italian participants but not Taiwanese peers [58]. Collectively, this suggests the need for additional analysis of the role of individual resilience in the SGM population to determine when it moderates relationships between stigma and health outcomes and when it does not.

Most studies of resilience in the SGM community focus on individual resilience only; however, there has been a call to integrate individual and community resilience in the study of the mental health of SGM people [41,58]. According to Meyer, exclusively focusing on individual resilience might lead to health disparities by implying that because one *can* be resilient to stressors, one *should* be resilient to stressors [32]. This assumption, in turn, leads to increased stress in the disadvantaged group as members focus their responses to a stressor on how they process it rather than focusing on society’s responsibility to protect the disadvantaged group from the stressor. Our finding that community resilience in the form of social support is a moderator in a way that individual resilience is not means that society, rather than the individual, has a major role to play in attenuating mental health issues among its SGM population.

In our study, participants who had higher community resilience scores were more likely to indicate that they had a friend or family member with whom they could discuss personal issues and by whom they felt supported and encouraged. They also reported always having someone to turn to for help and friends and family members who appreciated their qualities. Other research examining community resilience as a moderator of stigma in SGM populations is both limited and mixed. Some studies have found a moderating influence of community resilience, in the form of peer support, on mental health outcomes in the SGM community [44,45]. Other studies did not find a moderating effect on resilience. For example, community resilience did not moderate the impact of proximal and distal stress on suicide risk among gender minorities [59].

The broader body of research focused on the moderating role of community resilience in the context of suicidal ideation is also mixed [36,37,60,61]. In their systematic review, Johnson and colleagues determined that the moderating effect of social support on suicidal ideation was inconclusive but that there were enough research findings to suggest that the interaction may exist and to warrant further research in the area [36]. They also found support for the moderating effect on suicidal ideation of family support and support from a partner. Because findings about the role of community resilience as a moderator for suicidal ideation are mixed in the literature, additional research is necessary to understand the potentially powerful impact of community resilience. Our finding that community resilience moderated suicidal ideation among SGM people supports interventions to bolster community resilience and the need to study the links between such interventions and suicidal ideation in SGM people and more broadly.

As research continues, comprehensive, resilience-based, health-related interventions may help increase resilience among SGM people to improve mental health, especially suicidal ideation [62]. While Meyer warned against exclusively focusing on individual resilience, he did not recommend abandoning individual resilience research or interventions altogether. Rather, he suggested that individual and community resilience be considered as a continuum, with interventions focused on both types of resilience [32]. Individual resilience can be bolstered through interventions that increase cognitive coping, affect regulation, and refine problem-solving skills. Community resilience can be improved through peer support from SGM mentors, connecting with SGM-affirming support groups, and using SGM-tailored social media [63]. A study in China found that using SGM-tailored social media was associated with mental well-being through enhanced group membership and reduced SGM stigma [64]). Strong social ties developed through SGM-tailored social media may have the potential to build camaraderie and bolster community resilience among SGM people, especially in countries with limited protections and strong biases against SGM people. Interventions can additionally aim to increase both individual and community resilience. For example, *Positive action—Promoting change* is an empowered peer-group-based intervention founded to increase resilience among gender minority individuals in Italy through identity affirmation, self-acceptance, and group support [65].

Of course, none of this negates the more direct—and maybe more effective—pathway to target SGM people’s suicidal ideation and mental health outcomes connected to structural stigma: concerted efforts to minimize structural stigma placed upon people who are SGM. Structural stigma occurs through complex cultural and social processes; however, structural stigma targeting SGMs can also take the shape of formal societal decisions, memorialized through mechanisms like laws and policies, or even proposed laws and policies, to discriminate against people who are SGM. Structural stigma in the form of state-level exclusionary laws and policies can reflect discriminatory societal-level conditions or cultural norms within the state that influence the mental health of SGM people. At the same time, exclusionary laws and policies can shape societal-level conditions or cultural norms, exposing SGM people to increased discrimination [66]. Decision-makers should be aware of the added mental health risk to SGM people of anti-SGM legislation and work toward more inclusive rather than exclusive laws and policies.

### Limitations

Due to the cross-sectional nature of this study, causation cannot be determined. Because this was an online survey, there may be issues of self-report bias and self-selection bias. Our exclusive focus on state-level transgender sports bans in the US and not other adopted or proposed discriminatory laws and policies in the US and elsewhere may also be a limitation. However, our research was motivated by the recent increase in US states proposing anti-transgender laws and the potential impact of structural stigma on the mental health of SGM communities. Additionally, transgender sports bans are one limited aspect of structural stigma. Other forms of structural stigma, including other exclusionary laws and policies, societal-level conditions, and cultural norms that were not measured in this study, may have influenced the results. They may confound our analyses and may also account for variance in suicidal ideation. Precisely for this reason, future studies should examine the role of other forms of structural stigma in influencing suicide and suicidal ideation in the SGM population and the moderating role of resilience [67]. Future research is also needed to better understand the broader influence of other forms of structural stigma, such as the repel of Roe vs. Wade, legal threats to marriage equality, and laws restricting access to gender affirming healthcare on the health of SGM populations. Lastly, the number of participants who identified as transgender or gender non-binary was small. We could not conduct analyses to determine differences in outcomes between cisgender and transgender/non-binary participants even though research has shown differences in mental health outcomes between subsets of the SGM community [68].

## 5. Conclusions

SGM people familiar with structural stigma in the form of adopted or proposed transgender sports bans had higher suicidal ideation scores than those who were not familiar with the bans. While these sports bans target only a small portion of the SGM community, they have far-reaching impacts on the mental health of the greater SGM community. Decision-makers should consider the broader health impacts of these proposals on the SGM population. However, when anti-SGM laws and policies are in place, interventions to increase both community and individual resilience may attenuate some mental health impacts.

## Figures and Tables

**Table 1:** Sample characteristics for main predictor, moderators, covariates, and outcome.

Variable	# of Missing	Mean	SD
Suicidal ideation	10	18.36	10.57
Individual resilience	65	85.48	22.21
Community resilience	67	33.74	9.37
Age	10	38.56	15.72
	# of missing	N	%
Structural stigma	34		
Familiar		326	32.63
Not familiar		673	67.37
Sexual orientation	9		
Bisexual		465	46.97
Gay		224	22.63
Lesbian		160	16.16
Other		141	14.24
Gender identity	0		
Female		554	55.46
Male		316	31.63
Other		129	12.91
Race	0		
Black		89	13.22
White		498	74.00
Other races		52	7.73
Multiple races		34	5.05
Ethnicity	5		
Non-Hispanic		853	85.39
Hispanic, Spanish, Latinx		146	14.61
Education	5		
High school degree or less		299	29.93
Some college, no degree or associate degree		355	35.54
Bachelor or higher degrees		345	34.53
Marital status	5		
Divorced, separated, widowed		134	13.41
Married or unmarried couples		405	40.54
Single (never married)		460	46.05
Employment status	6		
Employed		484	48.45
Homemaker, retired, student		296	29.63
Unable to work		116	11.61
Unemployed		103	10.31
Income	6		
Less than $20,000		340	34.03
$20,000–$49,999		337	33.73
$50,000 or more		322	32.23

Abbreviation: SD = Standard deviation.

**Table 2:** Estimated coefficients, confidence intervals, and *p*-values of structural stigma, individual and community resilience, and suicidal ideation—univariate and multivariate linear regression models.

Variable	Estimate ^§^	95% CI	*p*-Value
Univariate moderation model I			
Structural stigma	4.72	(−0.41, 9.84)	0.0714
Individual resilience	−0.20	(−0.23, −0.16)	<0.0001
Structural stigma × Individual resilience	−0.002	(−0.06, 0.06)	0.9550
Univariate moderation model II			
Structural stigma	12.29	(7.44, 17.15)	<0.0001
Community resilience	−0.37	(−0.45, −0.30)	<0.0001
Structural stigma × Community resilience	−0.22	(−0.35, −0.08)	0.0018
Multivariate moderation model			
Structural stigma	9.96	(4.53, 15.40)	0.0003
Individual resilience	−0.15	(−0.19, −0.11)	<0.0001
Community resilience	−0.19	(−0.28, −0.10)	<0.0001
Structural stigma × Individual resilience	0.06	(−0.004, 0.13)	0.0664
Structural stigma × Community resilience	−0.31	(−0.47, −0.15)	0.0002

^§^ All estimates were adjusted by age, sexual orientation, gender identity, race, ethnicity, education, marital status, employment status, and income.

**Table 3:** Comparisons in suicidal ideation between participants with and without familiarity with structural stigma in the form of transgender sports ban legislation in low, medium, and high levels of individual resilience and community resilience.

Individual Resilience ^§^	Community Resilience ^§§^	Difference	95% CI		*p*-Value
Low	Low	6.46	(4.53,	8.40)	<0.0001
Low	Medium	3.59	(1.64,	5.54)	0.0003
Low	High	0.73	(−2.16,	3.61)	0.6215
Medium	Low	7.85	(5.85,	9.85)	<0.0001
Medium	Medium	4.98	(3.73,	6.24)	<0.0001
Medium	High	2.12	(0.22,	4.01)	0.0288
High	Low	9.24	(6.30,	12.19)	<0.0001
High	Medium	6.38	(4.44,	8.31)	<0.0001
High	High	3.51	(1.69,	5.32)	0.0002

^§^ Low = 63.51; medium = 85.70; high = 107.89. ^§§^ Low = 24.45; medium = 33.82; high = 43.19.

## Data Availability

Data are not available due to privacy.
